# α-Cyperone (CYP) down-regulates NF-κB and MAPKs signaling, attenuating inflammation and extracellular matrix degradation in chondrocytes, to ameliorate osteoarthritis in mice

**DOI:** 10.18632/aging.203259

**Published:** 2021-07-08

**Authors:** Huawei Zhang, Sunlong Li, Jiajie Lu, Jie Jin, Gaosheng Zhu, Libo Wang, Yingzhao Yan, Linjie He, Ben Wang, Xiangyang Wang, Huachen Yu

**Affiliations:** 1 Department of Orthopaedics, The Second Affiliated Hospital and Yuying Children’s Hospital of Wenzhou Medical University, Wenzhou 325000, Zhejiang Province, China; 2 Key Laboratory of Orthopaedics of Zhejiang Province, Wenzhou 325000, Zhejiang Province, China; 3 Department of Orthopaedics Surgery, Zhejiang Hospital, Hangzhou 310000, Zhejiang Province, China; 4 Department of Orthopaedics Surgery, Zhongshan Hospital, Shanghai 200032, China

**Keywords:** α-Cyperone, osteoarthritis, inflammation, extracellular matrix degradation

## Abstract

Inflammation and extracellular matrix (ECM) degradation have been implicated in the pathological process of osteoarthritis (OA). α-Cyperone is the main active component of the traditional Chinese medicine *Cyperus rotundus L*. In this study, we found that α-Cyperone abolished the IL-1β-induced production of inflammatory cytokines in isolated rat chondrocytes, such as cyclooxygenase-2 (COX-2), tumor necrosis factor alpha (TNF-α), interleukin-6 (IL-6) and inducible nitric oxide synthase (iNOS), in a dose-dependent manner (0.75, 1.5 or 3 μM). Also, the results showed that α-Cyperone downregulated the expression of metalloproteinases (MMPs) and thrombospondin motifs 5 (ADAMTS5), and upregulated the expression of type-2 collagen. Mechanistically, molecular docking tests revealed that α-Cyperone stably and effectively binds to p65, p38, extracellular signal-regulated kinase (ERK), and c-Jun N-terminal kinase (JNK). α-Cyperone inhibited NF-κB activation by blocking its nuclear transfer, and decreasing the phosphorylation of mitogen-activated protein kinase (MAPKs). In addition, *in vivo* studies based on a mouse model of arthritis showed that α-Cyperone prevented the development of osteoarthritis. Therefore, α-Cyperone may be a potential anti-OA drug.

## INTRODUCTION

Osteoarthritis (OA) is a degenerative joint disease that often occurs in the elder population [1], and mainly characterized by articular surface erosion, subchondral hyperosteogeny and synovitis. The disease has been linked to a variety of risk factors, including aging, joint overload, and obesity [2]. Moreover, several inflammatory mediators have been implicated in its pathological progression and development [3]. The most important inflammatory factor among them, interleukin-1 *β* (IL-1*β*), mediates downstream inflammation-related proteins and degradation of extracellular matrix (ECM), such as nitric oxide (NO), prostaglandin E2 (PGE2), matrix metalloproteinases (MMPs) and thrombospondin motifs (ADAMTS), and is involved in the occurrence of articular surface damage [4]. Moreover, these inflammation cytokines have also been detected in the cartilage, synovium, osteophyte or joint capsule fluids of osteoarthritis patients [5–7]. Therefore, inhibiting IL-1*β* or downstream inflammatory factors may be a potential approach for treating osteoarthritis.

Previous studies have affirmed IL-1*β*’s role in activating the NF-κB signaling pathway [8]. Particularly, NF-κB signaling plays a key role in promoting inflammation and catabolism, whereas IκBα binds to NF-κB to inhibit its activity in the cytoplasm. Pro-inflammatory cytokines such as IL-1*β*, lipopolysaccharide (LPS) and tumor necrosis factor α (TNF-α) have been shown to mediate IκBα degradation, by binding to the IL-1 receptor (IL-1R), thereby causing transfer of NF-κB into the nucleus. Consequently, there is a marked activation of inflammatory factors and damage to the matrix homeostasis, such as MMPs, ADAMTS and IL-6. In addition, IL-1*β*-mediated phosphorylation activates mitogen-activated protein kinase cascades (MAPKs), including p38, extracellular signal-regulated kinase (ERK) and c-Jun N-terminal kinase (JNK), which subsequently promotes chondrocytes inflammation and degradation of their ECM [9–12]. Therefore, inhibiting expression of the NF-κB and MAPKs signaling pathways may ameliorate osteoarthritis.

*Cyperus rotundus L*, a member of family Cyperaceae, is a valuable traditional Chinese medicine with worldwide distribution. Previous studies have shown that its underground rhizomes contain α-Cyperone (CYP), an active natural terpenoid that inhibits LPS-induced inflammation of BV-2 cells through inhibition of the NF-κB signaling pathway [13]. Furthermore, CYP was also found to exert its anti-inflammatory efficacy in cardiac microvascular endothelial cells by inhibiting expression of the MAPKs pathway [14]. However, despite its action in inhibiting inflammation across a wide variety of tissues, CYP’s protective role on OA has not been fully investigated. Therefore, this study sought to explore CYP’s protective effects against IL-1*β*-induced inflammatory response and ECM degradation, and to further explore its potential mechanisms in rat chondrocytes. Meanwhile, we established OA mice models to detect CYP’s protective role in the progression of OA.

## RESULTS

### Effects of CYP on viability of rat chondrocytes

**Figure 1:**
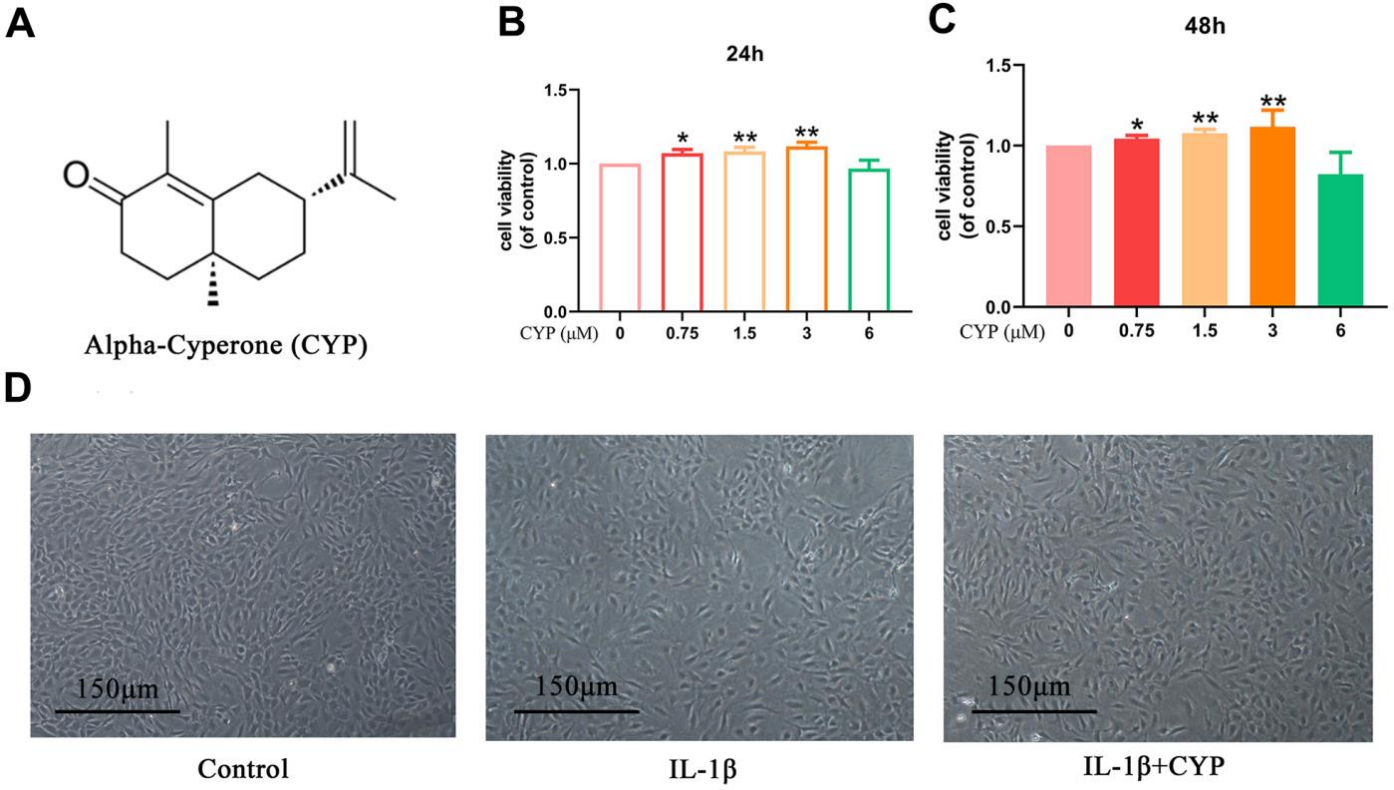
**Effects of CYP on survival rate of chondrocytes.** (**A**) Structural chemical formula of CYP. (**B**, **C**) CYP’s cytotoxicity of in an increasing concentration (0, 0.75, 1.5, 3, 6 μM) on chondrocytes as measured by CCK-8 assay after a 24 or 48-hour incubation. (**D**) Morphology of chondrocytes as observed under a microscope following treatment by IL-1β (10 ng/ml) combined with or without CYP (3 μM) after 24 hours of incubation (scale bar: 150 μm). Data presented are means ± S.D. of five independent experiments. * *p* < 0.05 and ** *p* < 0.01 vs. the control group, n=5.

The chemical structural formula of CYP is shown in Figure 1A. We determined CYP’s cytotoxicity on rat chondrocytes using the CCK-8 kit at a specified gradient concentration (0, 0.75, 1.5, 3 or 6 μM) for 24 or 48 hours. Results demonstrated showed that various CYP concentrations did not exert significant cytotoxicity on chondrocytes (Figure 1B, 1C). Therefore, we selected CYP concentrations of 0.75, 1.5 or 3 μM for use in subsequent experiments. Morphology and density of rat chondrocytes under different conditions (control, IL-1*β* and IL-1*β*+CYP) are shown in Figure 1D.

### Effects of CYP on inflammatory mediators in IL-1*β*-induced rat chondrocytes

**Figure 2:**
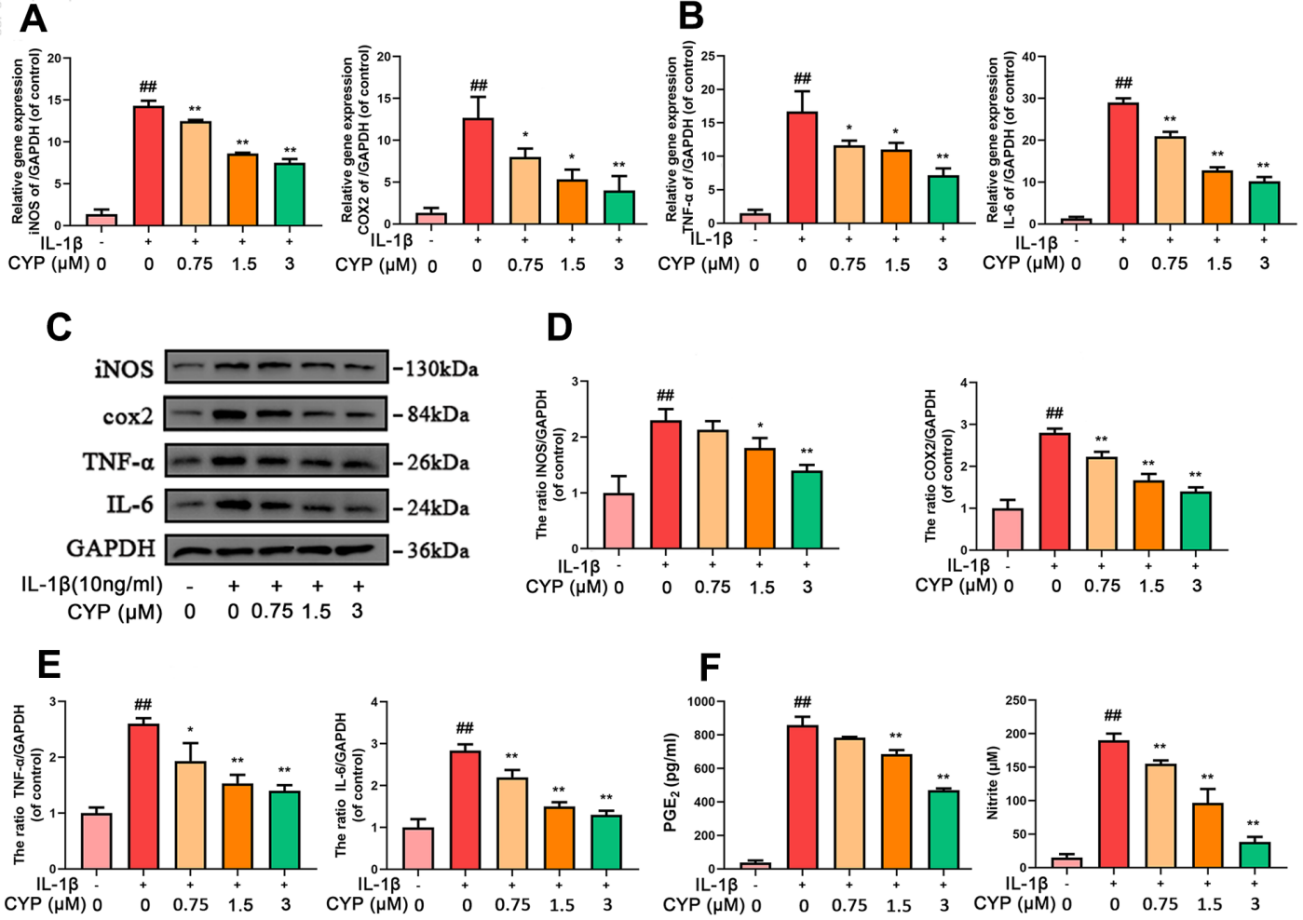
**CYP inhibits inflammation in chondrocytes induced by IL-1β (10 ng/ml).** Expression of INOS, COX-2, TNF-α and IL-6 genes were determined by real-time PCR (**A**, **B**). Levels of INOS, COX-2, TNF-α and IL-6 inflammatory mediators in rat chondrocytes were detected by western blot assay (**C**) and quantified by Image Lab software (**D**, **E**). Levels of PGE2 and Nitrite as measured by ELISA and Griess reagent method (**F**). Data are presented as means ± S.D. ## means *p* < 0.01 *vs.* the control group and ** *p* < 0.01, * *p* < 0.05 *vs.* the IL-1β alone group, n=5.

We explored the effect of CYP on levels of mRNA and protein expression of inflammatory cytokines (INOS, COX-2, TNF- α and IL-6) using qRT-PCR and western blot assay, respectively. Results indicated that CYP inhibited expression of inflammation-related mRNAs up-regulated by IL-1*β* (10 ng ml^-1^) in a dose-dependent manner (0.75, 1.5 or 3 μM) (Figure 2A, 2B). Similarly, CYP downregulated expression of inflammatory proteins in a dose-dependent manner (0.75, 1.5 or 3 μM) (Figure 2C–2E). However, no statistical significance was found between the group treated with a concentration of 0.75 μm CYP and the group treated with IL-1*β* alone for the protein expression of INOS (*p* = 0.315). Moreover, we found that CYP inhibited IL-1*β*-induced production of NO and PEG-2 in a concentrate-dependent manner (0.75, 1.5 or 3 μM) (Figure 2F), although it failed to achieve statistical significance at the concentration of 0.75 μM for PGE2 (*p* = 0.063). All results revealed significant differences among IL-1*β* alone, 1.5 and 3 μM groups (*p* < 0.05). Overall, these results indicated that CYP suppresses downstream inflammatory factors induced by IL-1*β* in a dose-dependent manner.

### Effects of CYP on ECM synthesis and degradation in IL-1*β*-induced rat chondrocytes

**Figure 3:**
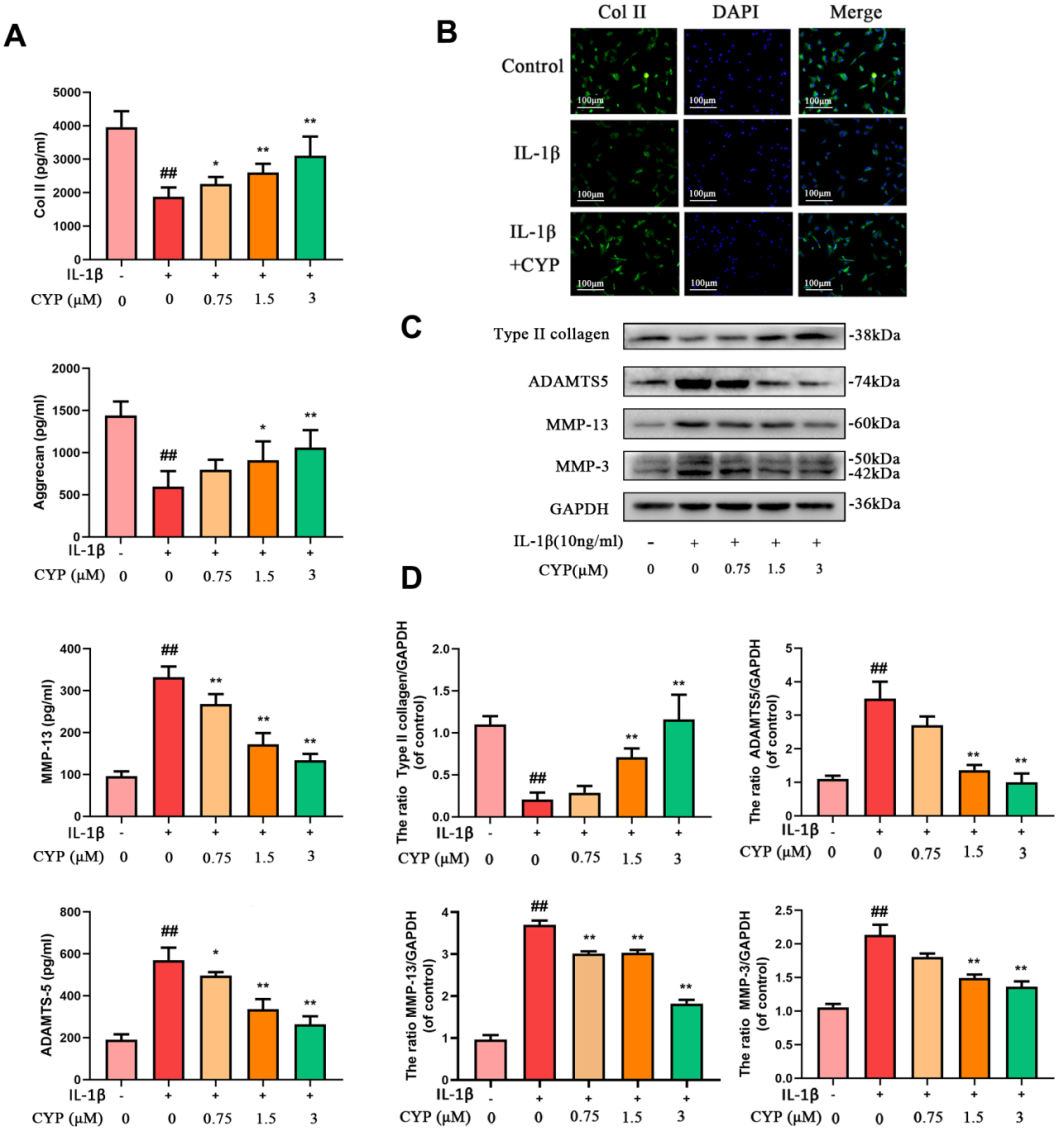
**Effects of CYP on IL-1β induced ECM degradation in chondrocytes.** Levels of type II collagen, aggrecan, MMP-13 and ADAMTS5 were measured by ELISA (**A**). The type II collagen was assayed through cell immunofluorescence combined with DAPI staining for nuclei (scale bar:100 μm) (**B**). Expressions levels for type II collagen, ADAMTS5, MMP-13 and MMP-3 were detected by western blot assay (**C**) and quantified by Image Lab software (**D**). Data are presented as means ± S.D.## means *p* < 0.01 *vs.* the control group and ** *p* < 0.01, * *p* < 0.05 *vs.* the IL-1β alone group, n=5.

We used western blot assay, ELISA test and immunofluorescence analysis to evaluate the effect of CYP on IL-1*β*-induced ECM degradation. CYP upregulated type-2 collagen and aggrecan in a dose-dependent manner, especially at 1.5 and 3 μM (*p* < 0.05), relative to the IL-1*β*-stimulated group. Conversely, CYP downregulated MMP3, MMP13 and ADAMTS5 in a concentration-dependent manner (0.75, 1.5 or 3 μM), especially at 1.5 and 3 μM (*p* < 0.01), compared to the IL-1*β*-stimulated group (Figure 3A, 3C, 3D). Meanwhile, immunofluorescence staining of collagen II across the control, IL-1*β* and IL-1*β* + CYP (3 μM) treatment groups showed that CYP inhibited the down-regulated effect of IL-1*β* on collagen II expression in chondrocytes (Figure 3B). Overall, these data suggest that CYP suppresses IL-1*β*-induced ECM degradation in rat chondrocytes.

### Molecular docking results

**Figure 4:**
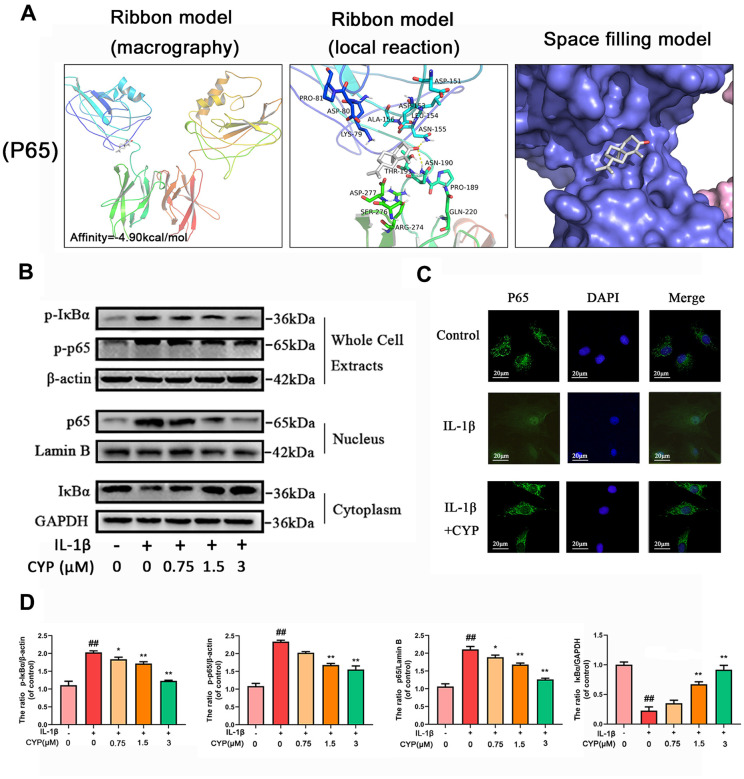
**Effect of CYP on NF-κb activation induced by IL-1β (10 ng/ml).** (**A**) Molecular docking results of CYP with p65 revealed that CYP was embedded in the binding pockets of proteins. Hydrogen bonds were built between CYP and ASN-155 and ASN-190. Expression levels of p-IκBα and p-p65 in whole chondrocyte extracts, p65 in nucleus and IκBα in cytoplasm were detected by western blot assay (**B**) and quantified by Image Lab software (**D**). (**C**) Nuclear translocation of p65 was detected through immunofluorescence combined with DAPI staining for nuclei (scale bar: 20 μM). Data presented are means ± S.D. ## means *p* < 0.01 *vs.* the control group and ** *p* < 0.01, * *p* < 0.05 *vs.* the IL-1β alone group, n=5.

**Figure 5:**
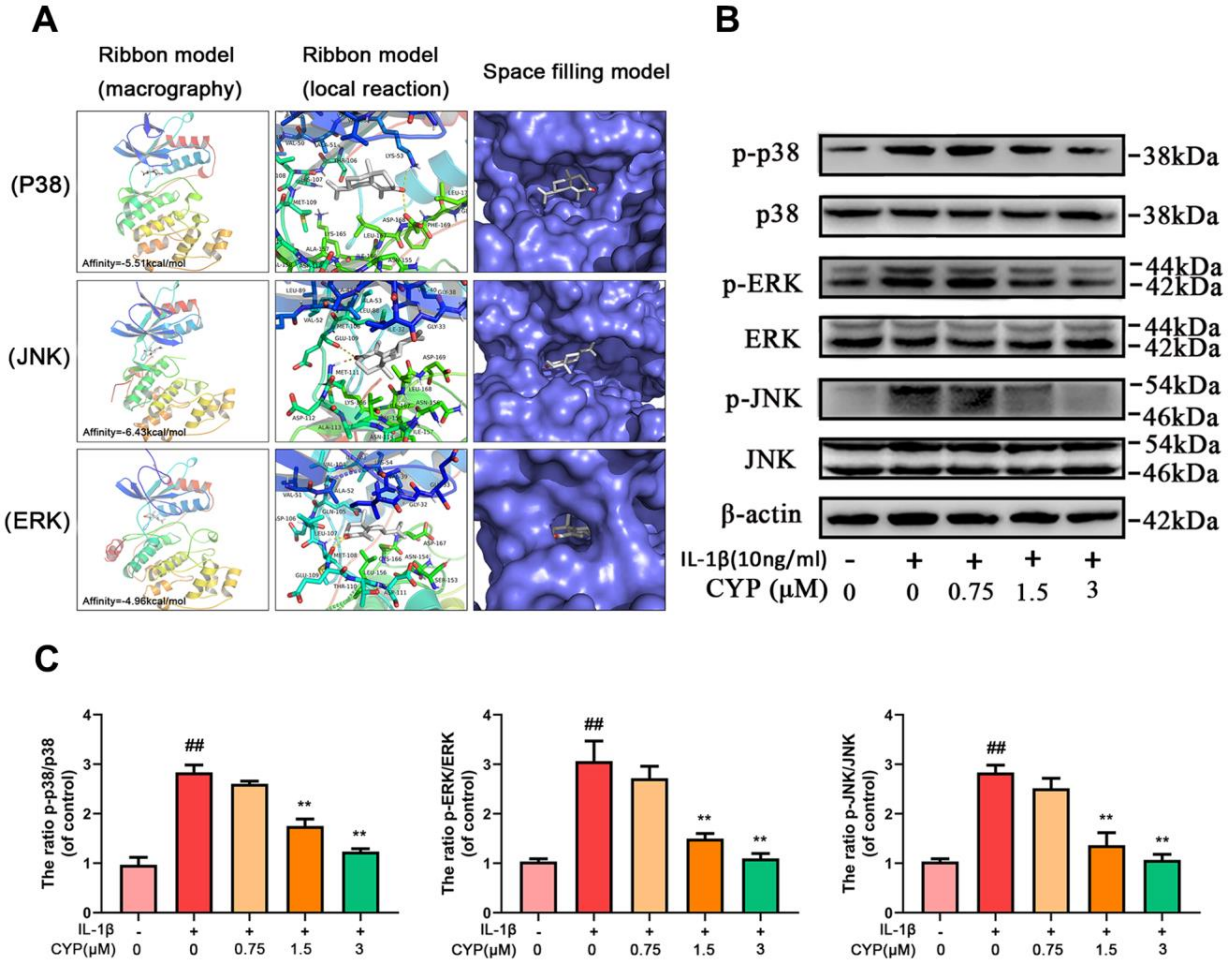
**Effect of CYP on MAPK activation induced by IL-1β (10 ng/ml).** (**A**) Molecular docking results of CYP with p38, JNK and ERK showed that CYP was embedded in the binding pockets of proteins. Hydrogen bonds were built between CYP and LYS-53, ASP-168, GLU-109, MET-111, LEU-107 and MET-108. Expression levels of p-p38, p38, p-ERK, ERK, p-JNK and JNK were detected by western blot assay (**B**) and quantified by Image Lab software (**C**). Data presented are means ± S.D. ## means *p* < 0.01 *vs.* the control group and ** *p* < 0.01, * *p* < 0.05 *vs.* the IL-1β alone group, n=5.

Ligands play a regulatory role by binding to protein pockets. To evaluate whether CYP participates in regulation of the NF-κB and MAPKs signaling pathways, we used molecular docking to analyze the binding affinity between CYP and pockets of receptor protein [15–17]. We performed several docking experiments with protein and CYP using Autodock software and visualized the results in PyMOL. Results showed that CYP achieved some stable hydrogen bonds with the binding pockets of p65 (ASN155 and ASN190, -4.90 kcal mol^-1^), p38 (LYS53 and ASP168, -5.51 kcal mol^-1^), JNK (MET111 and GLU109, -6.43 kcal mol^-1^) and ERK (MET108 and LEU107, -4.96 kcal mol^-1^). Moreover, our space-filling model showed that CYP is well embedded in the protein binding pockets (Figures 4A, 5A).

### CYP inhibits degradation of IκBα and activates IL-1*β*-induced NF-κB signaling pathways in rat chondrocytes

IL-1*β* induces phosphorylation of IκBα and NF-κb, causes degradation of IκBα and nuclear transfer of NF-κB and inducing expression of related inflammatory factors. To explore the mechanism through which CYP inhibits expression of NF-κB, as well as phosphorylation, we used western blot assay to measure protein levels of NF-κB and IκBα in the whole cell, NF-κB in the nucleus, and IκBα in the cytoplasm (Figure 4B, 4D). The results showed that CYP downregulated phosphorylated IκBα and NF-κB in the whole cells in a concentration-dependent manner (0.75, 1.5 or 3 μM), upregulated IκBα in the cytoplasm, and downregulated p65 in the nucleus. Moreover, Immunofluorescence staining of p65 in chondrocytes across the control, IL-1*β* and IL-1*β* + CYP (3 μM) treatment groups revealed that CYP inhibited transfer of p65 into the nucleus (Figure 4C). Taken together, these results indicate that CYP exerts an inhibitory effect on IκBα degradation and activation of NF-κB in rat chondrocytes.

### CYP inhibits expression of IL-1*β*-induced MAPKs pathway in rat chondrocytes

MAPKs are crucial proteins that activate inflammation and ECM degradation. On the other hand, IL-1*β* induced phosphorylation of ERK, JNK and p38, thereby activating expression of subsequent inflammatory cytokines. We used western blotting to explore the effect of CYP on MAPKs pathway, and found that it inhibits activation of this pathway in a concentration-dependent manner (Figure 5B, 5C). In addition, levels of p-p38, p38, p-JNK, JNK, p-ERK and ERK expression decreased with increase in CYP concentration, particularly at 1.5 and 3 μm (*p* < 0.01). Overall, these results showed that CYP inhibits expression of the MAPKs signaling pathway.

### CYP ameliorates OA progression *in vivo*

**Figure 6:**
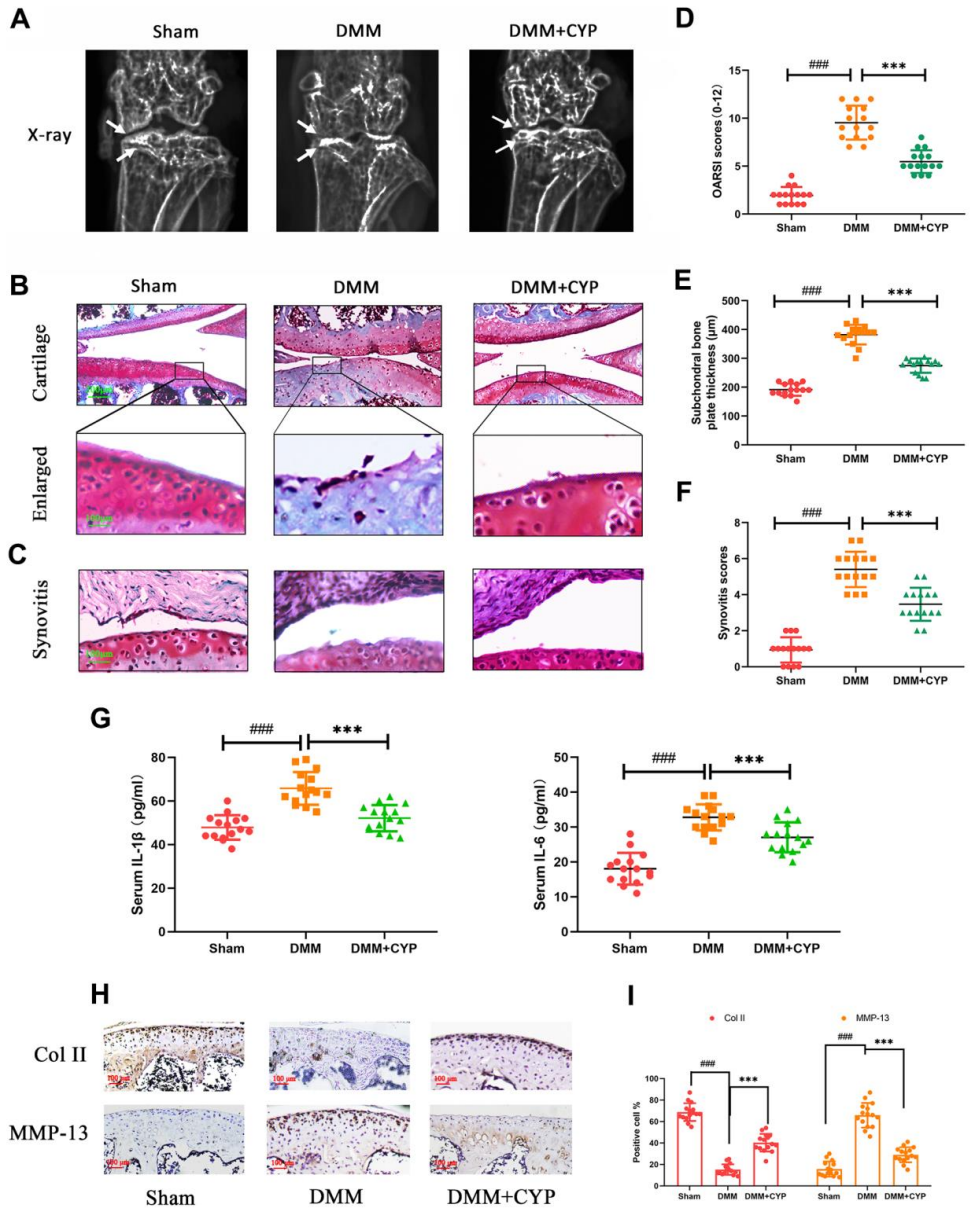
**CYP ameliorates knee joint degradation of OA in a surgery-induced mouse DMM model *in vivo*.** (**A**) Digital X-ray images showing the degree of degradation in the mouse knee joint across the three experimental groups. White arrows indicate the narrow space of joint and the formation of abnormal osteophyte. (**B**, **C**) Representative safranin O-fast green (S-O) staining of the cartilage and synovitis across the three experimental groups (scale bar: 200 μm in cartilage and 100 μm in enlarged). (**D**) OARSI scores of the cartilage across the three experimental groups. (**E**) Thickness of the subchondral bone plate across the three experimental groups. (**F**) Synovitis scores in the three experimental groups. (**G**) The amount of IL-1β and IL-6β in joint serum was detected by ELISA test. (**H**) Immunohistochemical staining of collagen II and MMP-13 expression in the cartilage samples (scale bar: 100 μm). (**I**) The percentages of collagen II and MMP-13 positive cells in each section were quantified by Image Pro Plus. Data are presented as means ± S.D. ^###^ denotes p<0.01 vs. the sham group while *** indicates p<0.05 vs. the DMM group, n=15.

To determine whether CYP ameliorates the development of osteoarthritis, mice were subjected to DMM surgery and randomly assigned into sham group, vehicle group and CYP treatment groups. Eight weeks after the operation, the amount of IL-1*β* and IL-6 in joint serum was detected by ELISA test and knee joints histological sections were obtained from all mice for immunohistochemical and histological staining analysis. The data showed that IL-1*β* amount in joint serum was 47,87 ± 5.643 on sham, 65.80 ± 7.495 on DMM and 52.13 ± 6.034 on DMM+CYP treatment group. Meanwhile, IL-6 amount was 18.29 ± 4.615 on sham, 32.36 ± 3.433 on DMM and 27.00 ± 4.438 on DMM+CYP treatment group (Figure 6G). Significant differences were found among groups (p < 0.05). results of revealed that mice in vehicle group developed symptoms of OA such as stenosis of articular cavity and abnormal osteophyte formation unlike those in the sham group as shown in Figure 6A. Of note, although CYP treatment developed some symptoms of OA, they were relatively milder compared to the vehicle group. The chondrocyte tissue was subjected to Safranin O staining to assess joint degradation, and the severity of OA was analyzed using OARSI scores, subchondral bone thickness scores and synovitis scores system. The results shown in Figure 6B, 6C, indicate that in the sham group, the joint surface was smooth and intact, and the chondrocytes showed normal staining. In contrast, the articular surface of vehicle group showed obvious pathologic changes, including apparent chondrocyte hypocellularity, massive proteoglycan loss and bone hyperplasia beneath the articular surface. Moreover, destruction of articular cartilage in the CYP treatment group was significantly milder than that in the vehicle group. In terms of scores (Figure 6D–6F), the vehicle group had the worst OARSI score of 9.533±1.767, subchondral bone plate thickness score of 382.0±33.85 and synovitis score of 5.40±0.985. The sham group had the best OARSI score of 1.933±0.883, subchondral bone plate thickness score of 191.3±21.0 and synovitis score of 0.933±0.703. For the CYP treatment group, the OARSI score was 5.467±1.187, subchondral bone plate thickness score was 274.6±24.69 and that of synovitis score was 3.467±0.915. These scores were significantly different between groups (p<0.05). In addition, immunohistochemical staining for collagen II and MMP-13 were performed, and results revealed that collagen II positive-staining cells were 68.73±7.96, 15.20±4.75 and 40.27±7.96 among sham, DMM and DMM+CYP treatment group. Conversely, MMP-13 positive-staining cells were 15.73±6.80, 65.80±11.07 and 29.0±6.77 among sham, DMM and DMM+CYP treatment group (Figure 6H, 6I). These values were significantly different between groups (p<0.05). In general, these results suggest that CYP alleviates the occurrence and development of OA by preventing chondrocyte ECM destruction and the inflammation of knee joint tissues.

## DISCUSSION

Osteoarthritis, a chronic orthopedic disease that causes bone and joint degeneration in joints with high load and range of motion such as knee or hip joint, predominantly affects the female elderly population [18]. A previous survey showed that 50% of people over the age of 65 suffer from osteoarthritis [19], a trend that poses a great challenge to public health [20]. In recent years, many physiopathologic factors have been linked with occurrence and development of osteoarthritis. To date, however, an effective treatment therapy for the disease is lacking [21–23]. Although non-steroidal anti-inflammatory drugs (NSAIDs) have been widely used in clinical treatment of osteoarthritis, they can only temporarily relieve pain and do not delay disease progression. Moreover, they have been associated with several side effects, key among them frequently damage to the cardiovascular system [24]. Therefore, it is imperative to develop novel therapeutic agents that can effectively inhibit degeneration of articular cartilage in osteoarthritis.

CYP is an active substance in the rhizome of *Cyperus rotundus L*, and reportedly alleviates inflammation by down-regulating COX-2 and PGE-2 via the NF-κB signaling pathway [25]. However, only a few studies have evaluated CYP’s protective effect on lesions of knee joint in OA. Therefore, the present study sought to investigate CYP’s anti-inflammatory effects in osteoarthritis. Previous studies have shown that NF-κB plays a crucial role in promoting cell growth, cellular immune responses and inflammation. The main role of IκBα in the cytoplasm is to cover the nuclear localization fragment of p65, thereby inhibiting transfer of p65 from the cytoplasm to the nucleus and prevent it from interacting with a specific sequence to induce inflammation. Degradation of IκBα by IL-1*β*-induced phosphorylation causes activation of p65 and triggers production of inflammatory mediators, such as INOS, COX-2, TNF-α and IL-6. NO catalyzed by INOS or TNF-α [26, 27], is a well-known inflammatory mediator, which not only inhibits synthesis of type II collagen but also upregulates MMPs [28]. Previous studies have shown that MMP-13 plays a crucial role in the MMPs family [29], inducing degradation of proteoglycans and type II collagen [30]. In addition, MMP-3 assists a degradation effect of MMP-13 on ECM. On the other hand, Prostaglandin E2 (PGE2), one of many prostaglandins (PG), is a metabolite of arachidonic acid cyclooxygenase and a key inflammatory mediator. Functionally, PGE2 induces production of MMPs and ADAMTS [31]. Among the ADAMTS family, ADAMTS-5 is the major enzyme that cleaves proteoglycan [32]. Normal chondrocyte metabolism is maintained by the support from type II collagen and proteoglycan [33]. However, degradation of type II collagen or proteoglycan induces apoptosis of chondrocytes [34]. Furthermore, apoptotic cells do not produce proteoglycan or type II collagen, but secrete proteolytic enzymes that further destroy the ECM [35, 36]. Overall, a series of inflammatory mediators downstream of NF-κB, including TNF-α and IL-6, play a vital role in promoting occurrence and development of OA. Based on this, we hypothesized that inhibiting the aforementioned inflammatory mediators can ameliorate damage to the cartilage’s extracellular matrix and effectively suppress OA. Results of the present study showed that CYP could significantly down-regulate INOS, IL-6, TNF-α and COX-2 induced by IL-1*β* at both protein and mRNA levels, as well as suppress synthesis of ADAMTS-5, MMP-3 and MMP-13. Taken together, these results suggest that CYP may be playing an anti-inflammatory role by blocking activation of p65 in OA.

**Figure 7:**
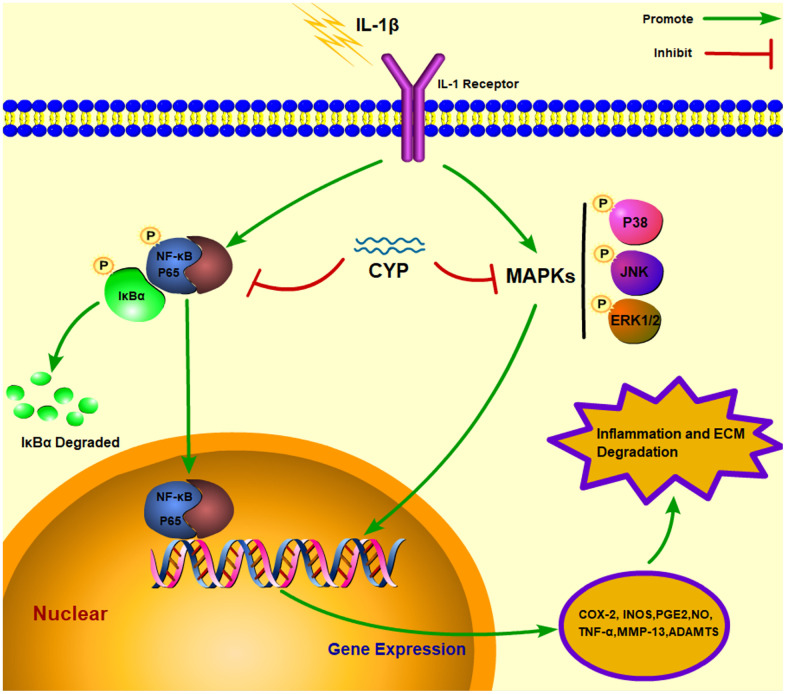
Schematic representation of CYP’s interaction with the NF-κb and MAPK signaling pathways in an IL-1*β* induced rat chondrocyte.

MAPK cascades are vital transducers of signals from the cell surface to the interior of the nucleus [37]. Specifically, it regulates a variety of important cellular physiological courses, such as cell division, differentiation and adaption [38, 39]. Previous studies have shown that MAPKs, including p38, JNK and ERK1/2, enhance process of osteocyte proliferation [40], tumor metastasis [41] and inflammatory disease [42]. Meanwhile, the MAPK signaling pathway has also been implicated in occurrence and development of osteoarthritis [11, 12, 43]. IL-1*β* induces phosphorylation of p38, JNK and ERK1/2, which further induces expression of COX-2 and PGE2, followed by that of MMPs and ADAMTSs. These inflammatory mediators destroy the extracellular matrix and cause knee joint degeneration [44–46]. Besides, MAPK has also been associated with activation of the NF-κB pathway, which also up-regulates related inflammatory factors that mediate the occurrence of OA. In the present study, results of the western blot assay showed that CYP can effectively block expression of p38, JNK and ERK1/2, thus delaying the pathological process of OA and preventing knee joint degeneration. The underlying mechanism of CYP action in blocking of the NF-κB and MAPK signaling pathways, as well as the interaction between molecules and ligands are described in Figure 7.

Results of molecular docking showed that CYP bound to the amino acid residues in the regular ligand-binding sites p65, p38, JNK and ERK, filled the binding pockets of these proteins and blocked their activation. We found specific building of stable hydrogen bonds between p65, p38, JNK and ERK and CYP, and these were accompanied by significantly high bond energy, which was -4.90 kcal mol^-1^ for p65, -5.51 kcal mol^-1^ for p38, -6.43 for JNK and -4.96 kcal mol^-1^ for ERK. Although CYP may also regulate other related key proteins upstream, such as the activation of SIRT1 suppresses NF-κB pathway as previously reported [47], our results revealed a clear and direct interaction between CYP and p65, p38, JNK and ERK.

To simulate the pathological mechanism of OA *in vivo*, DMM mouse model has been recommended due to its similarities with human joint damage [48]. The DMM mouse model’s articular cavity exhibited reduced chondrocytes, abnormal hyperosteogeny and destruction of ECM. Results from our histological staining and X-ray examination revealed significant improvement in joint degeneration in CYP-treated mice, meanwhile, immunohistochemical staining result also showed a prominent protective effect by CYP in cartilage matrix, in line with *in vitro* studies. Taken together, these findings indicated that CYP exerts a protective effect which alleviates OA-induced joint damage.

In summary, our data demonstrated that CYP significantly ameliorates IL-1*β*-induced inflammation and ECM degradation in rat chondrocytes. The underlying mechanism involves blocking activation of the NF-κB and MAPKs signaling pathways. Molecular docking revealed that CYP has high affinity for P65, P38, JNK and ERK. Notably, CYP’s protective effects were also observed in a DMM-induced OA mouse model. Taken together, these findings indicate that CYP is a potential therapeutic agent for treating OA.

## MATERIALS AND METHODS

### Ethics statement

Investigation has been conducted in accordance with the ethical standards and Declaration of Helsinki, and according to the Guidelines for the Care and Use of Laboratory Animals of the National Institutes of Health and has been approved by the Animal Care and Use Committee of Wenzhou Medical University (ethic code: WYDW2020-0160).

### Reagents and antibodies

Alpha-Cyperone (CYP) (purity >98%) and recombinant rat IL-1*β* were obtained from MedChemExpress (Monmouth Junction, NJ, USA) and PeproTech (Rocky Hill, NJ, USA), respectively. Primary antibodies against aggrecan, ADAMTS5, collagen II, MMP-3, *β*-actin, Lamin B1 and GADPH were obtained from Abcam (Cambridge, UK), whereas those antibodies against iNOS, COX-2, TNF, IL-6, MMP-13 were purchased from Proteintech Group (Wuhan, China). Primary antibodies against p65, p-p65, p38, p-p38, IκBα, p-IκBα, JNK, p-JNK, ERK and p-ERK were obtained from Cell Signaling Technology Sigma Aldrich (St Louis, MO, USA). Primary antibodies against p65, type II collagen and MMP-13 during immunofluorescence and immunohistochemical staining were obtained from Cell Signaling Technology Sigma Aldrich (St Louis, MO, USA). Goat anti-rabbit IgG (7074) and goat anti-mouse IgG (7076) were purchased from Cell Signaling Technology Sigma Aldrich (St Louis, MO, USA), while Alexa Fluor®488 labeled Goat anti-rabbit IgG (H + L) secondary antibodies were acquired from Bioworld (Dublin, OH, USA). 4′,6-Diamidino-2-phenylindole (DAPI) and Type II collagenase were purchased from Beyotime (Shanghai, China) and Abcam (Cambridge, UK), respectively, while all cell culture reagents were acquired from Gibco (Grand Island, NY, USA).

### Isolation and culture of primary rat chondrocytes

Procedures for extraction of primary rat chondrocytes were approved by the Animal Care and Use Committee of Wenzhou Medical University. Briefly, articular cartilage was excised from underlying bone and connective tissues of 7-day-old male rats [49]. The cartilage tissues were cut into 1×1×1 mm^3^ pieces, washed three times with phosphate-buffered saline (PBS), then incubated for 4-6 hours with type II collagenase (Sigma-Aldrich, St. Louis, MO, USA) at 37° C. The digested cartilage tissue was centrifuged at 1000 rpm for 3 minutes, the supernatant removed using a micropipette, and the residue resuspended in DMEM/F-12. The chondrocyte culture medium comprised 10% Fetal Bovine Serum (FBS) and 1% penicillin and streptomycin antibiotics. The chondrocytes were resuscitated, distributed in several Petri dishes, then cultured at 5% carbon dioxide at 37° C. The cells were allowed to grow to a density of 80-90% in the culture plate, then eluted with 0.25% trypsin-EDTA. The chondrocytes were collected then equally plated in a 10 cm^2^ petri dish at approximately 1 × 10^5^ cells ml^-1^. Changes of chondrocyte culture medium were performed every 1-2 days.

### Animal models

A total of 45 eight-week-old male C57BL/6 wild-type (WT) mice were purchased from the Animal Center of Chinese Academy of Sciences (Shanghai, China). The mice were kept in a cage, maintained at a temperature of 20-26° C, 45-70% humidity, 24-hours cyclic light, and supplied with abundant clean food and water. All the experimental and mice handling procedures, including feeding and treatment, were performed in accordance to the Declaration of Helsinki, the Guidelines for the Care and Use of Laboratory Animals of the National Institutes of Health, and the Animal Care and Use Committee of Wenzhou Medical University (ethic code: WYDW2020-0160). To establish an OA mouse model, we adopted a procedure for surgical destabilization of the medial meniscus (DMM) as previously described [48]. Briefly, mice were anesthetized with 4% chloral hydrate, then a 1.5 cm incision was made in the knee near the patellar tendon. The mice’s knee joint capsule was exposed and the medial meniscotibial ligament cut off using a microsurgical instrument. Care was taken to ensure no damage was caused to the lateral meniscotibial ligament during the operation. For mice in the sham group, we performed arthrotomy on their knee joint, but without incising the medial meniscotibial ligament. The mice were randomly distributed into sham, vehicle and CYP treatment groups with 15 in each group.

### Experimental design

We performed *in vitro* experiments to evaluate CYP’s anti-inflammatory effects. Briefly, rat chondrocytes were pretreated with IL-1*β* (10 ng ml^-1^) with or without different concentrations of CYP (0.75, 1.5 or 3 μM). Chondrocytes in the control group were subjected to no intervention, except regular replacement of culture medium, and the chondrocytes in this group collected after incubation of 24 hours. To investigate the activation effect of IL-1*β* on NF-κB and MAPKs signaling pathways, we first stimulated for 2 hours, then extended this time to 24 hours to allow monitoring of functional changes, such as expression of inflammation and ECM markers.

We also performed *in vivo* experiments, after DMM surgery. Here, we intraperitoneally injected mice in the CYP treatment group with a daily dose of 10 mg kg^-1^ CYP for 8 weeks. The CYP was dissolved in DMSO and the injection administered at 8:00 a.m. once a day. Mice in the vehicle group were intraperitoneally injected with corresponding amounts of normal saline. All mice were sacrificed, eight weeks after the operation, their joint fluid was extracted and the expression of IL-1*β* in the serum was detected by ELISA test. Followed that, their articular cartilage tissue samples were collected for radiographic and histological analyses.

### CCK-8 assay

We investigated CYP’s cytotoxic effect on rat chondrocytes using the cell counting kit-8 (CCK-8; Dojindo Co, Kumamoto, Japan). Briefly, primary chondrocytes were cultured in a 96-well plate for 24 hours, then incubated with different concentrations of CYP (0, 0.75, 1.5, 3, 6 μM) for 24 or 48 hours respectively. After a predetermined time point, 100 μl of DMEM/F12 and 10 μl CCK-8 reagent were added to each well followed by a 2-hour incubation in a calorstat. Finally, absorbance of each hole was read out using a spectrophotometer (Thermofisher) at 450 nm wavelength. Each experiment was replicated at least five times.

### Griess reagent and ELISA test

The content of NO in the petri dish was determined using the Griess reagent as previously described [50], while the amount of PGE2, collagen II, aggrecan, MMP-13 and ADAMTS5 in the supernatant of cell culture medium were measured using commercial ELISA kits (R&D Systems, Minneapolis, MN, USA). Meanwhile, *in vivo* experiment, the amount of IL-1*β* and IL-6 in the rat knee capsule was measured by ELISA kits. All procedures were performed according to the manufacturer’s instructions with each experiment replicated five times.

### Gene expression analysis

Total RNA was extracted from rat chondrocytes, following treatment of IL-1*β* (10 ng ml^-1^) and CYP with a certain concentration gradient (0.75, 1.5 or 3 μM), using the TRIzol reagent (Invitrogen). Approximately 1000 ng RNA was reverse transcribed into complementary DNA (cDNA), then used for quantitative real-time polymerase chain reaction (qRT-PCR), targeting iNOS, COX-2, TNF-α, and IL-6 genes. Primers were designed using the NCBI Primer-Blast Tool, and were as follows; COX-2 (F) 5′-GAGAGATGTATCCTCCCACAGTCA-3′ (R) 5′-GACCAGGCACCAGACCAAAG-3′; iNOS (F) 5′-TTTCTCCACGTTGTTGTTAA-3′, (R) 5′-TCAGGCTTGGGTCTTGTT-3′; IL-6, (F) 5′-AGTTGCCTTCTTGGGACTGATGT-3′, (R) 5′-GGTCTGTTGTGGGTGGTATCCTC-3′; TNF-α (F) 5′-GCGTGTTCATCCGTTCTCTACC-3′, (R) 5′-TACTTCAGCGTCTCGTGTGTTTCT-3′. qRT-PCR reactions were performed in a total volume of 10 μl, comprising 5 μl of 2 × SYBR Green Master Mix, 0.25 μl of each primer and 4.5 μl of diluted cDNA. Amplifications were performed in a CFX96 Real-Time PCR System (Bio-Rad Laboratories, Hercules, CA, USA). Cycle threshold (Ct) values were normalized to GAPDH, while calculations for relative mRNA level of each target gene were performed using the 2^-ΔΔCt^ method.

### Protein extraction

When chondrocytes in the medium had grown to 80%, we added 0.05% trypsin to the cell contents then collected the cells by centrifugation. Total proteins were extracted using the RIPA lysis buffer, supplemented with 1 mM phenylmethanesulfonyl fluoride (PMSF), whereas nuclear and cytoplasmic proteins in the cell lysates were isolated using a Nuclear and Cytoplasmic Protein Extraction Kit (Beyotime, Shanghai, China). Thereafter, lysates were subjected to ultrasound, on the ice, centrifuged at 12000 rpm for 30 minutes at 4° C, and protein concentrations determined by BCA protein assay kit (Beyotime, Shanghai, China).

### Western blotting

Protein was detected using a Western blot assay. Briefly, an equal concentration of proteins (40 μg) was separated via sodium dodecyl sulfate-polyacrylamide gel electrophoresis (SDS-PAGE), then transferred to polyvinylidene difluoride membranes (BioRad, Hercules, CA, USA). The membranes were blocked with 5% no-fat milk for 2 hours, then incubated, overnight at 4° C, with specified concentrations of the following primary antibodies: iNOS (1:1000), COX-2 (1:1000), p65 (1:1000), p-p65 (1:1000), JNK (1:1000), p-JNK (1:1000), ERK (1:1000), p-ERK (1:1000), IκBα (1:1000), p-IκBα (1:1000), p38 (1:1000), p-p38 (1:1000), collagen II (1:1000), ADAMTS-5 (1:1000), MMP-3 (1:500), MMP-13 (1:1000), GADPH (1:5000), Lamin-B (1:5000) and *β*-actin (1:5000). The membranes were then incubated with secondary antibodies (Cell Signaling Technology, St Louis, MO, USA) at room temperature for 2 hours, washed 3 times with TBTS, then treated with Chemiluminescence Reagent Plus (Invitrogen). Finally, protein expression was detected by Image Lab 3.0 software (Bio-Rad).

### Immunofluorescence staining

Chondrocytes were first treated overnight with a serum-free medium, and implanted on coverslips in a six-hole plate. The chondrocytes were treated with 10 ng ml^-1^ IL-1*β* for 24 hours, with or without 3 μM CYP, washed 3 times with PBS, fixed with 4% paraformaldehyde for 15 minutes, then treated with 0.1% TritonX-100 at room temperature for 10 minutes. The samples were then transferred to a wet box device, sealed with 5% fetal bovine serum albumin at 37° C for 1 hour, rinsed with PBS, and incubated overnight with PBS-diluted antibody against p65 (1:200) and collagen II (1:200) in a humid chamber at 4° C. The following day, the coverslips were washed with PBS, then incubated at room temperature with fluorescein-labelled goat anti-rabbit IgG antibody (1:400) in the dark for 1 hour. The fluid from each well was extracted and labelled with 4,6-diamidino-2-phenylindole (DAPI) for 5 minutes. Five visual fields across each coverslip were randomly selected and observed by fluorescence microscope, in a darkroom, by observers blinded to the experimental groups.

### Molecular docking

We studied molecular docking targeting performed in p65, JNK, ERK and p38 with CYP [15–17, 51]. Briefly, we downloaded a PDB format file of each protein at PDB database (https://www.rcsb.org/) (ID of p65: 1LE9, JNK: 4IZY, ERK: 2Y9Q and p38:1OUK), then simulated protein modification and receptor-ligand docking using AutoDockTools (version 1.5.6). We then determined the minimum binding energy of the ligand to the protein binding pocket using default parameters, then visualized the docking images using PyMOL (version 2.20).

### Radiographic analysis

Mice in all the three groups were subjected to X-ray examination, 8 weeks after surgery, to assess knee joint degeneration, including arthrostenosis, cartilage surface calcification and osteophyte formation. Analysis was performed using a small animal X-ray machine (Kubtec Model XPERT.8; KUB Technologies Inc.), with suitable X-ray films acquired by the 50 kV and 160A parameter setting.

### Histopathologic examination

Mice across all three groups were subjected to histomorphometric analysis. Summarily, mice were sacrificed, their knee joint tissues surgically dissected, fixed in 4% paraformaldehyde for 24 hours then decalcified in 10% neutral TA solution for 1 month. The tissues were embedded in paraffin, cut into 5-μm thick sections using a microtome, then stained using safranin O-fast green (S-O). Morphology of the cartilage and osteocytes were analyzed under a microscope, by another group of blind-treated histologists, using criteria and scoring methods described by the Osteoarthritis Research Society International (OARSI) scoring system [52]. A score of 0 represents normal cartilage, 0.5 = loss of proteoglycan with an intact surface, 1 = superficial fibrillation without loss of cartilage, 2 = vertical clefts and loss of surface lamina (any % or joint surface area), 3 = vertical clefts/erosion to the calcified layer lesion for 1% to 25% of the quadrant width, 4 = lesion reaches the calcified cartilage for 25% to 50% of the quadrant width, 5 = lesion reaches the calcified cartilage for 50% to 75% of the quadrant width, 6 = lesion reaches the calcified cartilage for >75% of the quadrant width. Then, the sum scores (0-12) of medial femoral condyle and medial tibial plateau were calculated for the evaluation of articular cartilage destruction. Besides, the synovitis severity was graded using a scoring system as previously described [53], change of thickness in synovial lining cell layer was assessed on a scale of 0 to 3 (0 points = 1 to 2 cells, 1 point = 2 to 4 cells, 2 points = 4 to 9 cells and 3 points = 10 or more cells) and cellular density in the synovial stroma was assessed on a scale of 0 to 3 (0 points = normal cellularity, 1 point = slightly increased cellularity, 2 points = moderately increased cellularity and 3 points = greatly increased cellularity). In addition, subchondral cortical bone was defined as the cortical bone overlying the adjacent trabecular bone and bone marrow space in Safranin O stained sections [54], and the measurement of subchondral cortical bone thickness (μm) was performed by AxioVision software in our study.

### Immunohistochemical analysis

After dewaxing with xylene, the tissue embedded sections were rehydrated and the endogenous esterase was removed using 3% hydrogen peroxide. The sections were then treated with 0.4% pepsin (Sangon Biotech, Shanghai, China) in a 37° C incubator for 20 min for antigenic retrieval. Afterwards, the sections were blocked with 5% goat serum at room temperature for 30 min, then incubated overnight with primary antibody at 4° C. Finally, HRP-conjugated secondary antibody was used for incubation. Fifteen sections were randomly selected from each group, and quantitative analysis was performed using Image-Pro Plus Software 6.0.

### Statistical analysis

All experiments were performed at least 5 times. Data were statistically analyzed using SPSS version 21.0, and presented as means ± standard deviation (SD). Comparison among groups was performed using one-way analysis of variance (ANOVA), while mean separations were conducted Tukey's test. Nonparametric data were analyzed using the Kruskal-Wallis H test. Data followed by p<0.05 were considered statistically significant.
